# The interconnected relationships between middle ear bulla size, cavitation defects, and chronic otitis media revealed in a syndromic mouse model

**DOI:** 10.3389/fgene.2022.933416

**Published:** 2022-10-10

**Authors:** Juan M. Fons, Natalie J. Milmoe, Michael R. G. Dack, Leena Joshi, Hannah Thompson, Abigail S. Tucker

**Affiliations:** Centre for Craniofacial and Regenerative Biology, Fl27 Guy’s Hospital, King’s College London, London, United Kingdom

**Keywords:** ear development, congenital birth defect, branchio-oto-renal syndrome, cavitation, basal cell hyperplasia

## Abstract

High incidence of chronic otitis media is associated with human craniofacial syndromes, suggesting that defects in the formation of the middle ear and associated structures can have a knock-on effect on the susceptibility to middle ear inflammation. Patients with branchio-oto-renal (BOR) syndrome have several defects in the ear leading to both sensorineural and conductive hearing loss, including otitis media. 40% of BOR syndrome cases are due to *Eya1* haploinsufficiency, with mouse models affecting *Eya1*, mimicking many of the defects found in patients. Here, we characterize the onset, consequences, and underlying causes of chronic otitis media in *Eya1* heterozygous mice. Cavitation defects were evident in these mice from postnatal day (P)11 onwards, with mesenchyme around the promontory and attic regions of the middle ear space. This mesenchyme was still prominent in adult *Eya1* heterozygous mice, while the wild-type littermates had fully aerated ears from P14 onwards. MicroCT analysis highlighted a significantly smaller bulla, confirming the link between bulla size defects and the ability of the mesenchyme to retract successfully. Otitis media was observed from P14, often presenting unilaterally, resulting in hyperplasia of the middle ear mucosa, expansion of secretory cells, defects in the motile cilia, and changes in basal epithelial cell markers. A high incidence of otitis media was identified in older mice but only associated with ears with retained mesenchyme. To understand the impact of the environment, the mouse line was rederived onto a super-clean environment. Cavitation defects were still evident at early stages, but these generally resolved over time, and importantly, no signs of otitis media were observed at 6 weeks. In conclusion, we show that a small bulla size is closely linked to defects in cavitation and the presence of retained mesenchyme. A delay in retraction of the mesenchyme predates the onset of otitis media, making the ears susceptible to its development. Early exposure to OM appears to exacerbate the cavitation defect, with mesenchyme evident in the middle ear throughout the animal’s life. This highlights that permanent damage to the middle ear can arise as a consequence of the early onset of OM.

## Introduction

Middle ear inflammation [otitis media (OM)] is the main cause of mild-to-moderate hearing impairment in children worldwide (Acuin, 2002; Bhutta et al., 2017). OM is a complex, multi-factorial disease characterized by persistent inflammation of the middle ear mucosa. Acute episodes of OM in children are associated with bacterial infections. Acute OM can lead to recurrent episodes or chronic OM (persisting for more than 3 months), causing a build-up of fluid in the middle ear (OM with effusion) and perforation of the ear drum (chronic suppurative OM, CSOM). It is estimated that there are 31 million episodes of CSOM a year, causing significant hearing loss in 160 million individuals (Acuin, 2002; Schilder et al., 2016). In many cases, the ear is able to recover from OM. However, hearing loss as a result of OM results in delayed speech, language, and poor educational progress (Cai and McPherson, 2017). Untreated chronic OM can eventually cause erosion of the middle ear bones and surrounding temporal bone (auditory bulla), leading to permanent hearing loss. Implantation of ventilation tubes to combat OM is a common reason for surgery in children and is a substantial cost to healthcare services (Acuin, 2002; Monasta et al., 2012).

Although acute OM is a risk factor for developing middle ear effusion, many children develop chronic OM with effusion with no preceding history of acute OM, which suggests separate etiologies (Bhutta et al., 2017). Analysis of families has shown that there is a significant genetic component to recurrent and chronic OM (Hafren et al., 2012). The anatomy of the middle ear and eustachian tube, host immune status, innate mucosal defense, and pathogen virulence all play a role in the pathogenesis of the disease. Children with syndromes that affect the craniofacial region are particularly susceptible to chronic OM. These include 22q11 Deletion Syndrome, Down Syndrome, Turner Syndrome, Noonan Syndrome, and Ectodermal Dysplasia (Bhutta et al., 2017; Giese et al., 2020). Mouse models can be very useful in understanding the mechanisms underlying chronic OM in these syndromes and provide an opportunity to chart how the ear responds to OM over time (Bhutta et al., 2017).

Here we have focused on a mouse model of branchio-oto-renal syndrome. Branchio-oto-renal (BOR) syndrome (OMIM 113650), is an autosomal dominant disorder, with hearing loss observed in 93% of affected individuals and accounts for 2% of profoundly deaf children (Abdelhak et al., 1997; Hone and Smith, 2001). BOR patients can have defects in all three parts of the ear (inner, middle, and external), and patients can therefore suffer from conductive, sensorineural, or mixed hearing loss. Additionally, BOR syndrome patients have been shown to display chronic OM (Lindau et al., 2014; Wang et al., 2018) and have a reduction in the size or shape of the middle ear space (Smith, 2018). Approximately 40% of BOR patients have a causative mutation in the transcription factor *Eya1*, a homologue of the *Drosophila eyes absent* gene (Abdelhak et al., 1997; Matsunaga et al., 2007), with additional mutations associated with *Six1* and *Six5*, which bind to Eya1 (Smith, 2018).

Mouse models of BOR syndrome, *Eya1* *+/−* and *Six1* *+/−* heterozygous mice, exhibit postnatal middle ear defects and pathology, which likely contribute to the deafness phenotypes. *Eya1* heterozygous mice showed signs of inflammation in the middle ear cavity (Xu et al., 1999), while *Six1* heterozygotes had loose connective tissue around the ossicles and severe hearing loss (Zheng et al., 2003). Homozygote *Eya1* mice die before birth from a range of complications (Xu et al., 1999). The related gene, *Eya4*, when knocked out in mice, leads to early onset of OM, with effusions evidenced in mice from 2 weeks of age (Depreux et al., 2008). The middle ear cavities of *Eya4 −/−* mice retain mesenchyme at a stage prior to signs of otitis media, when the cavity should be predominantly air-filled (Depreux et al., 2008). Cavitation of the murine ear to create an air-filled space occurs postnatally in mice (Richter et al., 2010). Defects in postnatal cavitation of the middle ear lead to retention of mesenchyme in the cavity at late juvenile and adult stages and have been suggested to be the predisposing factor in the development of otitis media (Del-Pozo et al., 2019). Mesenchyme disperses around the edges of the growing middle ear cavity during postnatal development, suggesting a role for growth of the middle ear in successful cavitation (Piza et al., 1998; Del-Pozo et al., 2019). In keeping with this, smaller middle ear cavity sizes have been correlated with the retention of embryonic middle ear mesenchyme (Richter et al., 2010). Indeed, middle ear cavities in *Eya4 −/−* and *Six1* +/− mice were found to be smaller than the cavities of corresponding wild-type mice, implicating middle ear cavity size as a contributing factor to the pathologies observed in these mice (Zheng et al., 2003; Depreux et al., 2008). *Eya4* −/− mice additionally exhibit narrower eustachian tubes that open into the middle ear cavity at an abnormal position (Depreux et al., 2008); suggesting changes in the eustachian tube might also contribute to the development of chronic OM.

The occurrence of otitis media and related middle ear cavitation defects have not been investigated in detail in the *Eya1* mouse model. Here we analyze the timing of the onset of OM in *Eya1* heterozygous mice, identify mechanisms that may contribute to the high incidence of OM, and investigate the interactions between bulla size, retained mesenchyme, and OM.

## Materials and methods

### Mice

A generation of *Eya1*+/− mice has been previously described (Xu et al., 1999) with the strain name B6.129-Eya1 tm1Rlm/Mmucd (MGI 2150426). *Eya1* heterozygous mice were bred on a C57/Bl6 background and were culled using schedule 1 methods at a range of postnatal stages for comparison to wild-type littermates. *Eya1* homozygote mice die during embryogenesis prior to cavitation of the middle ear (Xu et al., 1999). Both male and female mice were used in the study. Antibiotic treatment from birth was provided by the addition of enrofloxacin (Baytril) to the drinking water at a final concentration of 0.25 g/l. Enrofloxacin is secreted into milk, allowing pups to acquire antibiotics during lactation as well as during drinking water (Del-Pozo et al., 2022). To assess the effect of the environment, mice were rederived via sperm transfer to a clean unit to assess the impact of the environment on the susceptibility to OM. In most cases, N = ears rather than N = mouse, as there were clear variations within the same mouse with regards to morphology and incidence of OM.

### MicroCT

Bulla volume, bulla internal airspace volume, and eustachian tube angle parameters were determined by microCT using a Scanco μCT50 (Scanco Medical, Zurich, Switzerland). Samples were scanned at 90 kV, 88 μA, with a 0.5-mm aluminum filter, 303 ms integration time, no averaging, and images were captured every 0.36° through 180° rotation. Bulla volume and eustachian tube parameters were calculated from whole-skull scans at a voxel resolution of 10 μm. Reconstructions and analyses were performed using Scanco software. Contours were drawn for both the inside of the bulla and the internal airspace of the bulla. For auditory bulla volume, the region of interest was taken by drawing a straight line from the tympanic notch at the base of the ear drum to the top outside edge of the roof of the bulla. Eustachian tube angle measurements were performed as described previously (Fuchs et al., 2015). Statistical analysis on the bulla was performed using unpaired one-tailed t-tests for bulla volume and bulla internal airspace, in order to test the hypothesis that the bulla was smaller in the hets. In both cases, Welch’s correction was included as the het mice were known to have a more variable phenotype compared to their wild-type littermates. For the eustachian tube angle analysis, an unpaired two-tailed *t*-test was used as the angles could increase or decrease. For the bulla volume, only the left ear was used for the analysis, while for the other measurements, both ears were used.

### Histology

After collection, samples were fixed in 4% paraformaldehyde (PFA) and decalcified in 0.5M EDTA pH = 8 for 14 days (p7), 20 days (p21), and 30 days (6w). After washing with PBS, heads were dehydrated in gradually increasing ethanol concentrations (30%, 50%, 70%, 90%, and 100%, 3 h per step). Heads were cleared in xylene (2–3 h) and embedded in paraffin (4–5 h). Sections were cut at 8 µm and stained with alcian blue, sirius red, and Ehrlich’s hematoxylin.

### RNAscope

For the analysis of *Eya1* expression, the RNAscope multiplex fluorescence assay (Advance Cell Diagnostics, ACD, a BioTechne brand) was performed following manufacturer’s instructions. The probes used were Mm Eya1 (Ref 430161-C1) and MmCdh1 (Ecad) (408651-C2). Cdh1 (Ecad) was used to outline the epithelium and as a positive control for the technique. For each stage, N = 3.

### Immunofluorescence

Sections were dewaxed 3 × 10 min in xylene and rehydrated in EtOH series (100%, 90%, 70%, 50%, and 30%) for a period of 2 min for each step. Antigen retrieval was performed in citric acid 0.01M at 90°C for 30 min. Blocking was achieved in PBS with 0.025% Tween20, 1% BSA, and 10% serum. After ON incubation at 4°C, the primary antibody was washed in PBT 0.025%, and a secondary antibody was incubated for 1 h at RT. After washing, the slides were mounted with fluoroshield. Primary antibodies were anti-acetylated tubulin 1/300 (Sigma-Aldrich, T7451), anti-Plunc1 1/200 (R&D systems, AF4274), and anti-K5 1/300 (Biolegend, PRB-160P), and secondaries were Goat anti-Mouse-568 1/500 (Invitrogen, A11004), Donkey anti-Sheep-488 1/500 (Invitrogen, A11015), and Donkey anti-Rabbit 647 1/500 (Invitrogen, A31573).

For the detection of PCNA 1/300 (Abcam, ab19166) and P63 1/400 (Abcam, ab124762), a secondary Goat anti-Rabbit biotin was used 1/800 (Dako, E0432) followed by Streptavidin-HRP (Abcam, ab64269). The color reaction was performed in TSA buffer (100 mM Borate buffer with 0.0003% hydrogen peroxidase) with Opal-570 1/300 (Akoya Bioscience, OP-001003) for 10min. DAPI was used to stain nuclei. For each genotype, N = 3.

## Results

### *Eya1* mice have retained mesenchyme linked to a high incidence of otitis media

**FIGURE 1:**
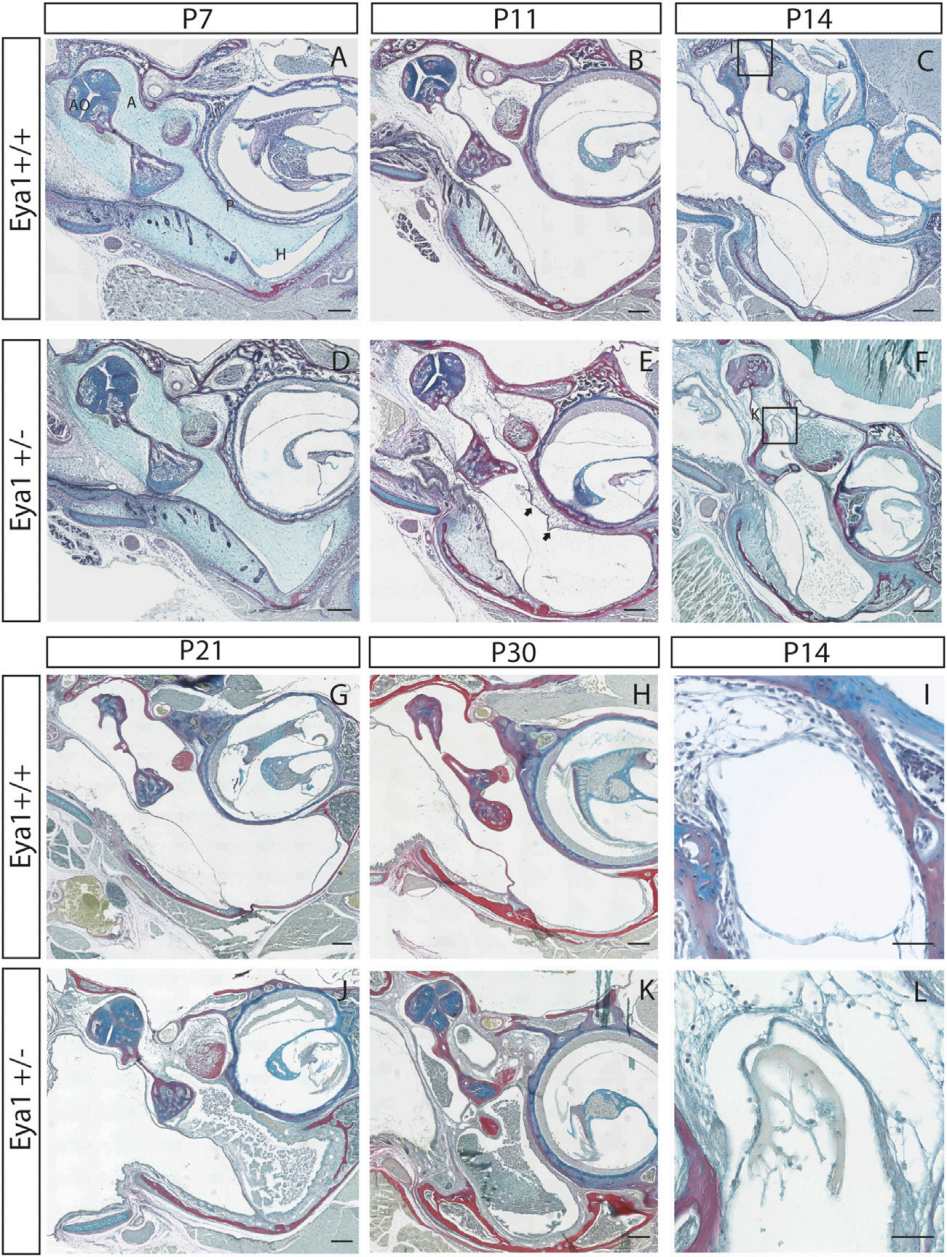
*Eya1* mice have retained mesenchyme linked to a high incidence of otitis media. *Eya1*+/+, **(A–C,G–I)** *Eya1*+/− **(D–F,J–L)** frontal trichrome-stained sections of the middle ear. **(A,D)** There is a potential slight delay in cavitation already evident at P7. **(B,E)** By P11 retained mesenchyme can be observed on the promontory of the middle ear in Eya1+/− mice [arrows in **(E)**], which is not present in wild-type mice at this stage. **(C)** In wild-type mice the middle ear has almost completed cavitation by P14 [highlight area shown in **(I)**]. **(F)** OM is observed in the mutants at P14 with effusion abutting the retained mesenchyme and debris dispersed throughout the middle ear [highlight area shown in **(L)**]. **(G,H,J,K)** Disease progresses in the mice as they age, characterized by thickened epithelium along the hypotympanum and build-up of exudate in Hets **(J,K)**. Juveniles shown are 30 days old **(H,K)**. [A, attic; P, promontory; H, hypotympanum. Scale bars in **(A–H,J,K)** set to 200 µm, scale bars in **(I,L)** set to 50 µm].

The onset of otitis media in the *Eya1* heterozygous mice was followed through a postnatal developmental series. At postnatal day (P) 7, the middle ear cavity is yet to cavitate and is filled with neural crest-derived mesenchyme (Richter et al., 2010). At this stage, a potential subtle difference was evident between the middle ears in *Eya1* het and wild-type (WT) mice (Figure 1A,D) (N = 6 WT and 6 *Eya1* het ears). By P11, the majority of the mesenchyme had retracted back in the WT mice, leaving an air-filled space in the hypotympanum (H) next to the eustachian tube (Figure 1B). At this stage, mesenchyme was still located around the ossicles in the attic (A) of the middle ear, with the head of the malleus and incus still embedded in tissue (Figure 1B). In the heterozygous mutants at this stage, the ossicles were similarly embedded in tissue, but mesenchyme was additionally observed around the promontory, which was clear of tissue in the WT (Figure 1E) (N = 6/8 Hets). At this stage, there was no evidence of otitis media in the middle ear. The extent of mesenchyme retention varied considerably in the individual mutants at this stage, ranging from ears where the extent of cavitation appeared as WT to those where large swaths of mesenchyme were still present in the hypotympanum (Supplementary Figure S1). The first signs of otitis media were observed in the mutant ears at P14 (5/8 ears) (Figure 1C,F,I,L). By this stage, the attic had cavitated in the WTs, leaving the ossicles free to vibrate (Figure 1C), but the mutants still had mesenchyme mesenchyme around the ossicles (N = 8/8 ears) (Figure 1F). Otitis media progressed in the mutants over time, with exudate build-up, and thickening of the epithelium along the hypotympanum (Figure 1G,H,J,K), following the described progression in other syndromic mutants (Fuchs et al., 2015; Tucker et al., 2018). Not all ears were equally affected, with some mice showing unilateral otitis media or none at all. We compared 7 *Eya1* mutants at P21 (i.e., after cavitation had completed in wild-type mice). Of these, 36% (N = 5/14) of heterozygotic ears did not exhibit otitis media. In all cases, these mutant ears did not display any retained mesenchyme in the bulla, which highlighted that the otitis media in these mice appeared to be triggered by the defect in cavitation.

### *Eya1* heterozygotes have smaller auditory bulla size

**FIGURE 2:**
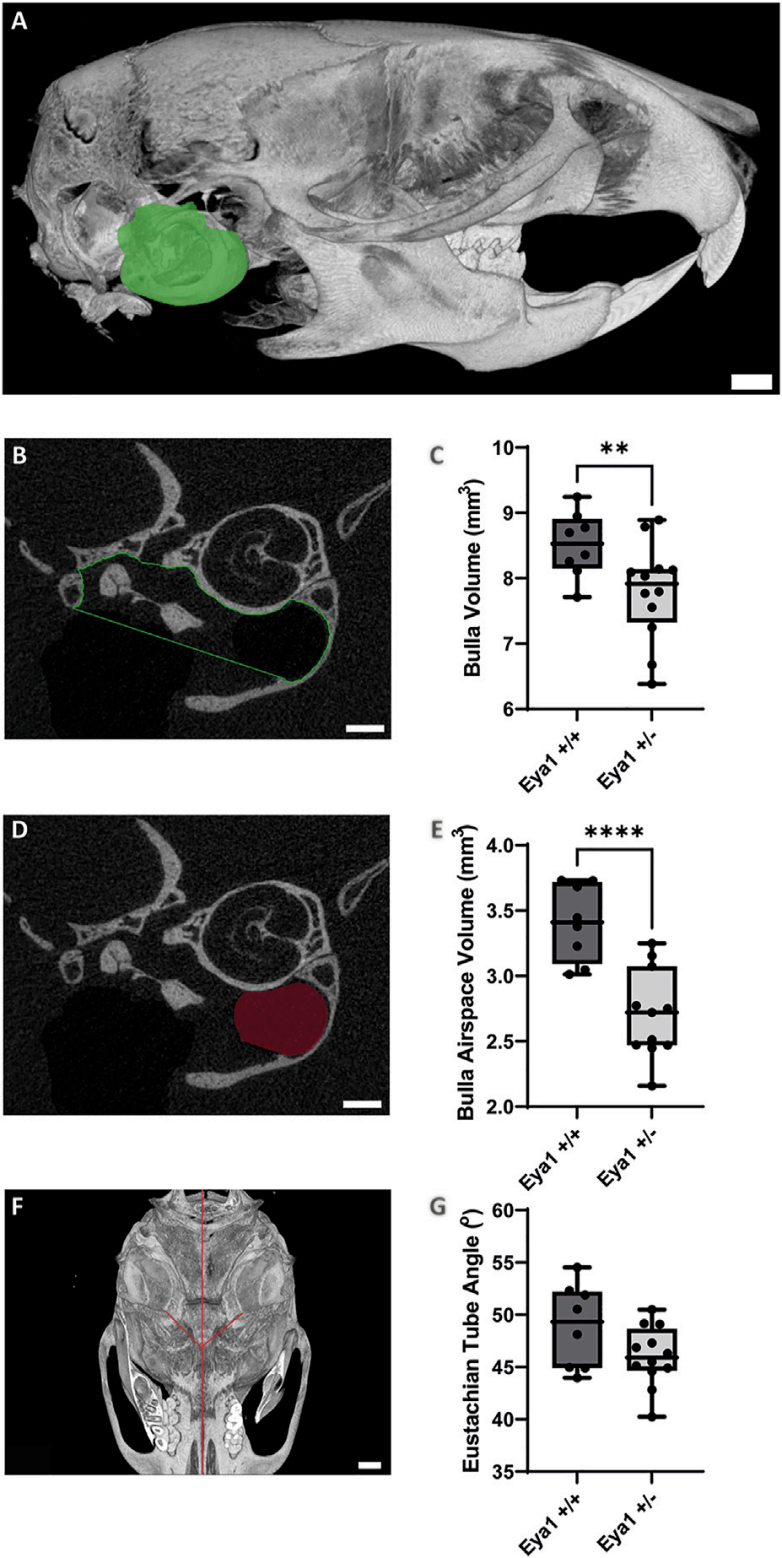
MicroCT analysis highlights smaller auditory bulla in *Eya1 het* mice. **(A)** Sagittal view of 3D-reconstruction of mouse skull showing segmented bulla volume in green (Scale bar = 1 mm). **(B)** Individual section of middle ear showing ROI selection for bulla volume measurements (Scale bar = 500 µm). **(C)** Box plot showing a significant reduction in bulla volume in *Eya1*+/− mice (LHS bulla) compared to wild-type mice (**p value = 0.0091). **(D)** Individual section of middle ear showing ROI selection for hypotympanum airspace volume measurements (Scale bar = 500 µm). **(E)** Box plot showing a significant reduction in hypotympanum airspace volume in *Eya1*+/− ears compared to wild-type ears (****p value = < 0.0001). **(F)** Ventral view of a 3D-reconstruction of mouse skill showing Eustachian tube angles in red from the middle ear cavity orifice to the midline of the skull (Scale bar = 1 mm). **(G)** Measurement of Eustachian tube angles from 3D-reconstruction skulls showing no significant difference between *Eya1*+/− and wild-type ears (*p* value = 0.0758).

Retained mesenchyme has been linked to a smaller auditory bulla (Richter et al., 2010) and several mouse models that develop otitis media possess smaller middle ear cavities (Hardisty et al., 2003; Depreux et al., 2008). The size of the auditory bulla was, therefore, assessed by creating a volume measurement of the inside of the middle ear from microCT scans in 6 week old mice (N = 4 WT mice (2 male/2 female) and 6 het mice (2 male/4 female) left hand bulla analyzed) (Figure 2A). The het ears were found to have a significantly smaller middle ear space compared to their WT littermates (Figure 2B,C) (*p* = 0.0091). Both male and female het mice had smaller auditory bulla, but the small numbers did not allow for statistical testing (Supplementary Figure S2A). Auditory bulla volume measurement requires an estimation of the border between the middle and external ear. As an alternative measure that did not rely on any subjective elements, we additionally measured the volume of the airspace evident in the hypotympanum of the ears. This can be visualized by dark areas on the CT scan (Figure 2B,D). Again, a highly significant difference was evident, with the het ears having a much smaller hypotympanum airspace than the wild types (N = 8 WT and 11 het ears, 1 het ear had no airspace and, therefore, no measurement was possible, both ears were analyzed to provide two data points for each mouse) (Figure 2E, Supplementary Figure S2B) (*p* < 0.0001).

### *Eya1* mice have normal eustachian tube angles

The eustachian tube (ET), a structure closely associated with middle ear function, is also implicated in the development of otitis media. Children are considered more susceptible to middle ear infections due to the horizontal positioning of their eustachian tubes (Daniel et al., 1982). Concurrent with the growth of cranial structures, the ET is pulled into an oblique position by adulthood, which has been proposed to be more effective for middle ear drainage. Mouse models with defects in the angle of the ET have OM, including *Eya4, Fbxo11jf/+*, and *Sh3pxd2b* mice (Hardisty et al., 2003; Depreux et al., 2008; Yang et al., 2011), while defects in ET muscles have also been linked to syndromic OM (Fuchs et al., 2015). Given these links, the angle of the eustachian tube was compared in 6-week-old mice using a modified methodology previously used to detect ET defects in *Sh3pxd2b*, *Chd7*, and *Df1* mice (Yang et al., 2011; Tian et al., 2012; Fuchs et al., 2015) (Figure 2F). No difference was observed in angle of the Eya1 hets and their wild-type littermates (N = 8 WT and 12 het ears, all ears were analyzed) (Figure 2G, Supplementary Figure S2C) (*p* = 0.0758).

### *Eya1* is expressed in the retracting epithelium of the middle ear and in the eustachian tube

**FIGURE 3:**
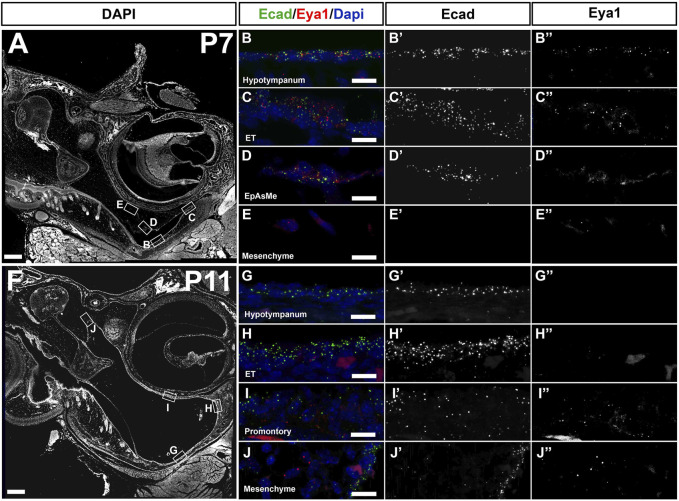
*Eya1* is expressed postnatally in the middle ear. RNAscope multiplex assay for *Ecad* in green and *Eya1* in red. **(A–E)** Postnatal day 7 (P7). **(F–J)** Postnatal day 11 (P11). *Eya1* is expressed (single dots mark mRNA molecules) in the epithelium of the hypotympanum **(B,B’’)**, eustachian tube (ET) **(C,C’’)** and in the epithelium associated mesenchyme (EpAsMe) **(D,D’’)** of the retracting mesenchyme. *Ecad* highlights the middle ear epithelium **(B’,C’,D’)**. *Eya1* shows very low to no expression in the retracting mesenchyme **(E,E’,E’’)**. At P11 **(F)**, *Eya1* is downregulated in the hypotympanum and ET epithelia **(G,G’’, H,H’’)** but remains in the promontory **(I,I’,I’’)** and retracting mesenchyme **(J,J’,J’’)**. *Ecad* remains on in the epithelium **(G’,H’,I’,J’)**. N = 3 for each stage. Scale bars: **(A,F)** = 200 µm; **(B–E)**, **(G–J)** = 12.5 µm.

*Eya1* has a complex expression pattern spanning the whole of embryonic ear development, from E9.5 to postnatal stages, with expression in the ectoderm, endoderm, neural crest, and mesoderm of the craniofacial region (Xu et al., 1997; Kalatzis et al., 1998; Amin and Tucker, 2006). To investigate the expression of *Eya1* postnatally in the middle ear, we used RNAscope, which allows a precise analysis of mRNA levels within a tissue section. The expression was compared to *E-cadherin* (green dots) in order to distinguish between the epithelium and the underlying mesenchyme in the ear. The expression was analyzed at P7 (Figure 3A–E), as cavitation was about to start, and at P11 (Figure 3F–J), a stage when a cavitation defect was already evident in mutants. *Eya1* mRNA (red spots in Figure 3) was identified in the ciliated epithelium of the hypotympanum and ET at P7 (Figure 3B,C), with a marked reduction in these areas by P11 (Figure 3G,H). In keeping with this, *Eya1* was enriched in ciliated cells of the lung respiratory track in the human protein atlas (https://www.proteinatlas.org/). Expression of *Eya1* was also observed in the epithelium lining the retracting mesenchyme with low levels in the underlying mesenchyme at both P7 and P11, suggesting a possible role in the retraction of the mesenchyme (Figure 3D,E,I,J). In accordance with previous reports, *Eya1* additionally showed postnatal expression in the inner ear, fitting with the role of *Eya1* in inner ear development and function and the sensorineural defects observed in some BOR patients (Supplementary Figure S3) (Kalatzis et al., 1998).

### Cilia, secretory cell, and proliferation changes are a consequence of otitis media

**FIGURE 4:**
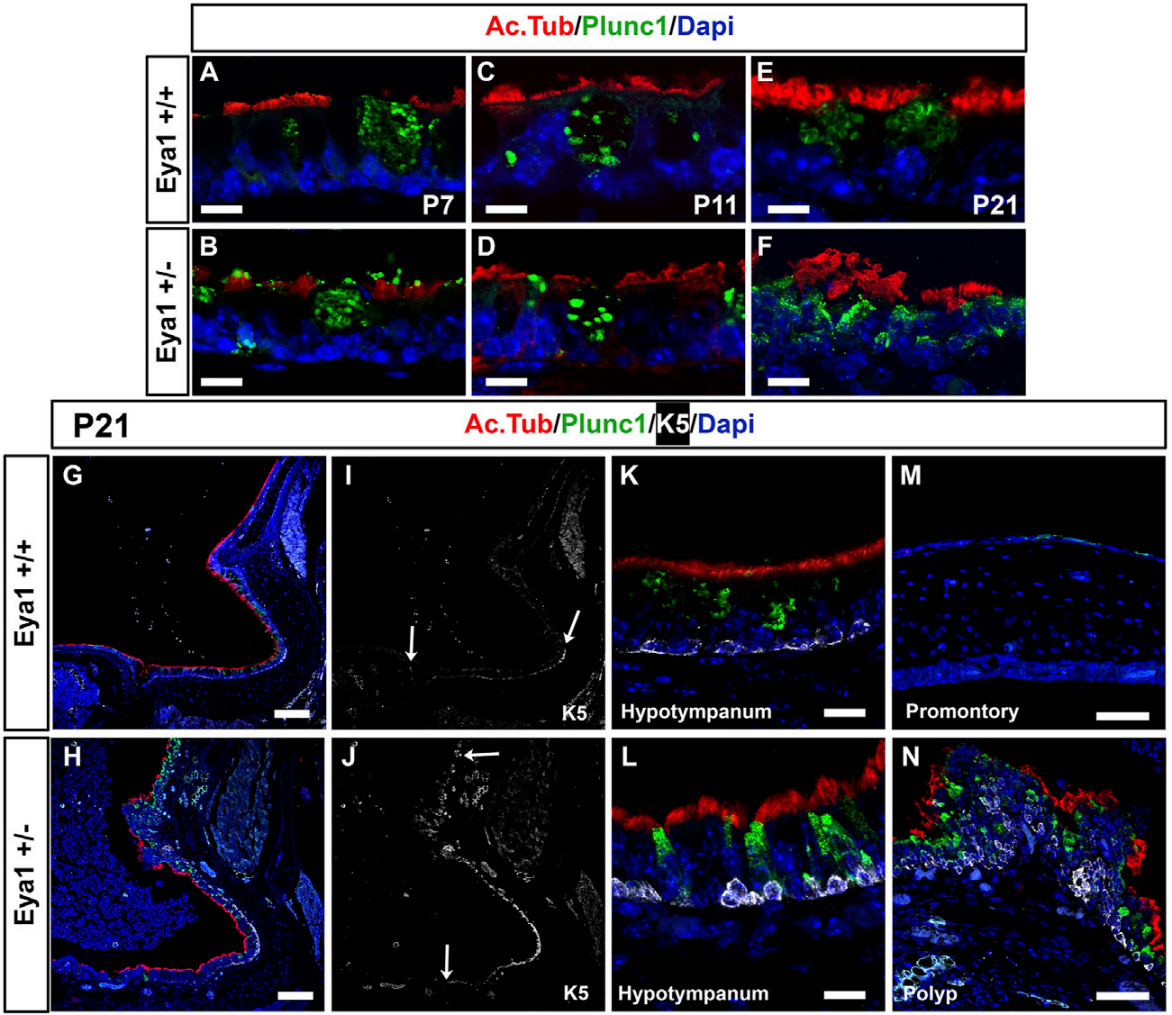
Defects in secretory and ciliated cells are a consequence of OM. Immunofluorescence for acetylated tubulin (red) to label ciliated cells, for Plunc1 (green) for secretory cells and keratin 5 (white) for basal epithelial cells. **(A,B)** P7. **(C,D)** P1. **(E–H)** P21. Wild-type littermates **(A,C,E,G,I,K,M)**. *Eya1* heterozygotes **(B,D,F,H,J,L,N)**. **(A–F,K,L)** Hypotympanum. **(M,N)** Promontory. **(A–D)** Secretory cells (green) and ciliated cells (red) appear normal at P7 and P11, before the onset of OM. **(E,F,K,L)** Secretory cells and ciliated cells have disrupted morphology in the hypotympanum by P21 after the onset of OM. **(K,L)** Keratin 5 (K5) positive cells (white) in the hypotympanum were associated with the basal epithelium, in both WT and hets. **(G,H)** The cilia (red) along the promontory becomes patchy compared to the wild-type, and the secretory cells, which are usually restricted around the eustachian tube, were expressed all the way along the promontory. **(I,J)** Same section as in **(G,H)** showing expression of Keratin 5 (white). Arrow at bottom of images indicates position of eustachian tube. **(I)** In the wild-type keratin 5 basal cells do not extend up towards the promontory. **(J)** In the heterozygous mice cells expressing K5 are expanded reaching the promontory in cases of OM (Compare top arrows, in I,J to indicate extent of labelled cells). **(M)** The wild-type promontory epithelium does not express keratin 5 or Plunc1. **(N)** Polyps were evident on the promontory in mutant mice with OM. These polyps contained K5 positive cells in suprabasal layers, with very disrupted cilia and an increase of secretory cells with aberrant morphology. N numbers: P7 n = 2 *Eya1*+/+, n = 3 *Eya1*+/-; P11 n = 2 *Eya1*+/+, n = 2 *Eya1*+/−. P21 n = 2 *Eya1*+/+, n = 3 *Eya1*+/−. Scale Bars: A-F,K,L = 12.5µm; G-J = 100 µm; M,N = 50 µm.

The eustachian tube (ET) and hypotympanum are covered in a thick lawn of cilia (Thompson and Tucker, 2013). The motile cilia of the middle ear and ET are important for the removal of mucus and debris from the cavity, with impaired mucociliary clearance linked to otitis media (Mata et al., 2014). Given the expression of *Eya1* in the hypotympanum and ET at P7, we examined whether the distribution of cilia and secretory cells was normal in the mutants at this stage, prior to the onset of otitis media. Acetylated tubulin (red) was used to mark the ciliated cells, while Plunc1 (green) was used to mark the secretory cells. Secretory cells and ciliated cells were both present in the epithelium at the opening of the eustachian tube at P7 and P11 in both the mutant and wild-type (Figure 4A–D). No noticeable difference was observed between the mutant and WT epithelium until the onset of otitis media, where significant thickening and disorganization of both ciliated and secretory cells were observed at P21 (Figure 4E,F,K,L). Instead of a single layer of secretory cells expressing Plunc1, double layers were observed as the epithelium started to stratify. Confirming the changes were a consequence of the otitis media; heterozygous mice without otitis media had a normal distribution of ciliated cells in the hypotympanum (data not shown). The promontory is usually negative for Plunc1, but secretory cells were observed to reach up to cover the promontory in cases of OM (Figure 4G,H). Changes in the epithelium of the promontory were even more pronounced with the formation of polyps that projected out into the middle ear cavity covered in dysmorphic cilia and secretory cells (Figure 4M,N). Staining for PCNA to label proliferating cells confirmed that the mutant ears with otitis media showed increased proliferation in the middle ear epithelium, particularly along the promontory at P21, while heterozygous ears without otitis media had very low levels similar to wild types (Supplementary Figure S4).

### Changes to basal cell markers as a consequence of otitis media

**FIGURE 5:**
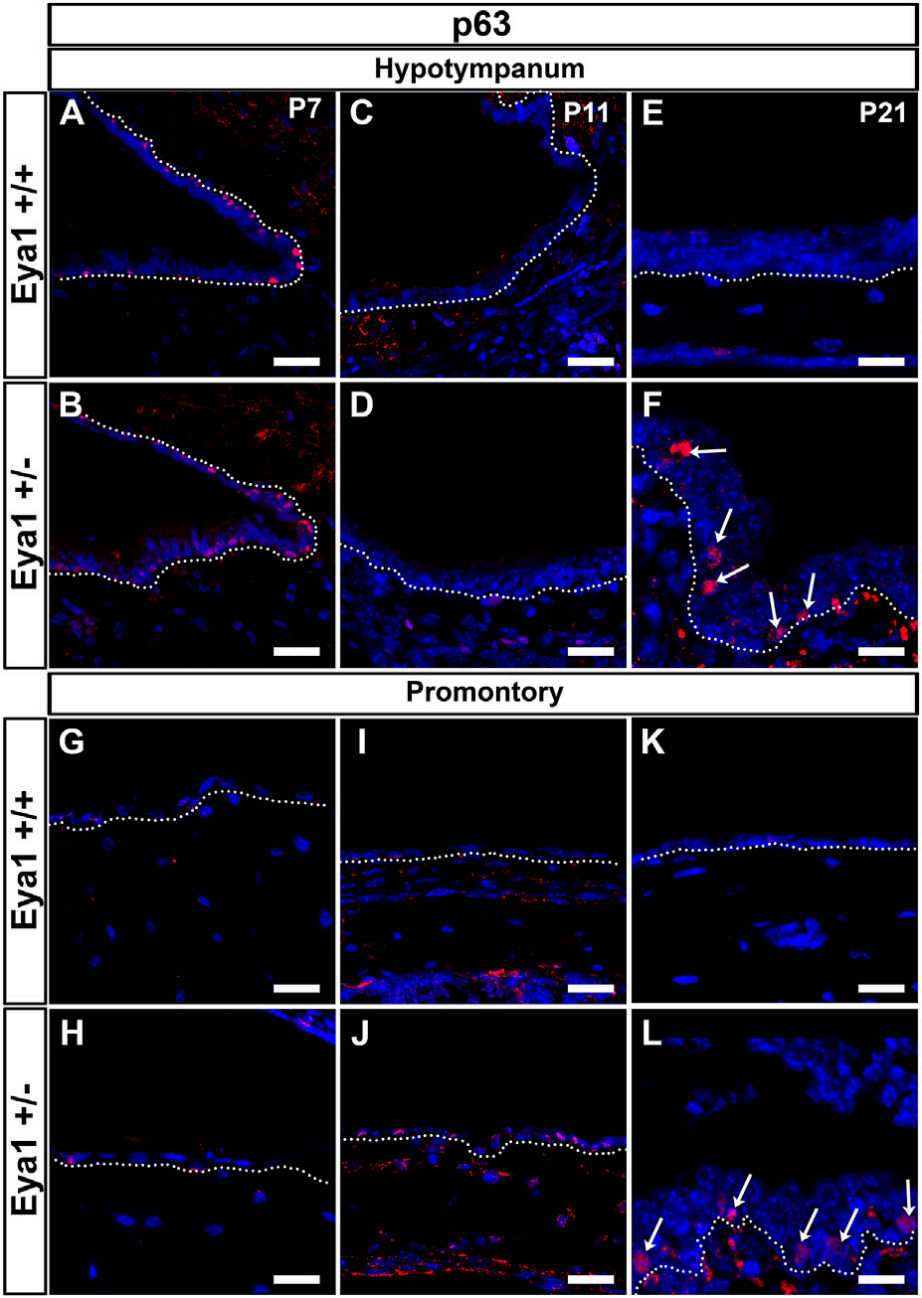
p63 is upregulated in cases of OM. p63 is expressed in the epithelium of the hypotympanum, at P7 in WT **(A)** and *Eya1* hets **(B)**, but it is downregulated at P11 as the epithelium matures **(C,D)**. In cases of OM, p63 was upregulated in this region [**(F)**, arrows] in contrast with the control situation **(E)**. The epithelium associated with the retracting mesenchyme at the promontory region at P7 **(G,H)** did not show p63 expression, and expression remained off in the epithelium of the promontory at later stages in wild types **(I,K)**. Expression was upregulated in the mesenchyme associated epithelium of *Eya1* hets at P11 during retraction **(J)** and in the epithelium of the promontory in cases of OM **(L)**, correlating with the presence of a hyperplastic stratified epithelium. N numbers: P7 n = 2 *Eya1*+/+, n = 3 *Eya1*+/−; P11 n = 2 *Eya1*+/+, n = 2 *Eya1*+/−. P21 n = 2 *Eya1*+/+, n = 3 *Eya1*+/−. Scale Bars = 25 µm.

Basal cell hyperplasia is a common phenotype associated with chronic secretory otitis media in patients based on histology (Tos, 1980). We therefore examined the expression of keratin 5 (K5) as a marker of epithelial basal cells. At P21 in wild-type littermates, K5 was expressed in the basal cells at the entrance to the eustachian tube and around the hypotympanum, but not along the promontory, agreeing with previous descriptions of wild-type mice (Tucker et al., 2018) (Figure 4I,M). In *Eya1* hets with OM, K5 expression extended all along the promontory (Figure 4J,N). In much of the promontory and hypotympanum, expression was still restricted to the basal cells, i.e., those in contact with the basement membrane (see Figure 4K,L), but in the polyps that extended out from the promontory, the K5 +ve cells were scattered throughout the epithelium and had lost their restriction to the basal layer (Figure 4N). *p63* is another marker of epithelial basal cells, that is essential for the proliferation of stem cells in stratified epithelium (Senoo et al., 2007). In wild-type mice, p63 was expressed in basal cells around the eustachian tube at P7, but expression was turned off as the tissue matured, with very little expression by P21 (Figure 5A,C,E). In contrast, p63 positive cells were not evident in the thin epithelium of the promontory in wild-type mice at P7-P21 (Figure 5G,I,K). The middle ear epithelium, therefore, has low levels of p63, which matches the low levels of proliferation in the mature middle ear. In contrast, as a consequence of otitis media, levels of p63 dramatically increased in the epithelium in both the hypotympanum near the ET (Figure 5B,D,F) and within the promontory (Figure 5H,J,L). As p63 is known to control proliferation in the skin, these changes are likely to trigger the hyperplasia observed in OM.

### The environment impacts the susceptibility to OM in *Eya1* het mice

To assess the impact of the environment on susceptibility, *Eya1* heterozygous mice were provided with antibiotics in their drinking water from birth. However, no reduction in the incidence of OM, was observed (9/10 hets with signs of OM). This may reflect the timing of treatment, the dosage provided, or the antibiotic given. As a more robust alternative, the *Eya1* line was rederived by sperm transfer in a clean environment to limit the impact of microbial infection. At P11, the majority of *Eya1* het mice on the clean side showed evidence of retained mesenchyme (N = 6/8 ears (4 mice) examined at this stage) (Supplementary Figure S1). However at 6 weeks , at an age when the majority of the *Eya1* hets on the conventional side had chronic otitis media, no evidence of otitis media was observed (N = 22/22 *Eya1* het ears) (Supplementary Figure S5). Of these mice, only one ear showed mild signs of retained mesenchyme (N = 1/22 ears). The environment therefore plays a big role in the development of otitis media, and defects in middle ear retraction can resolve over time in these mice but only in the absence of an ear infection.

## Discussion

In keeping with the observation of otitis media in BOR patients, high levels of chronic otitis media were observed in the majority of *Eya1* heterozygous ears when kept on the conventional side of the animal facility. The high susceptibility to otitis media in the *Eya1* mice was closely linked to the retained mesenchyme in the middle ear, with the defect in the mesenchymal retraction preceding any signs of OM. Some mutant ears underwent normal cavitation and did not display any signs of otitis media. In these cases, it is hypothesized that the bulla reached a large enough size to ensure mesenchymal retraction. A large bulla would be predicted to physically stretch out the mesenchyme, pulling the mesenchyme back until it lined the edges of the bulla. In support of this idea, the volume of mesenchymal cells in both humans and the opossum has been shown to stay constant during cavitation and just becomes redistributed within the bulla space as it grows (Piza et al., 1996; Piza et al., 1998).

Cavitation occurs postnatally in mice, while in humans the middle ear mesenchyme is mainly removed *in utero*, with around 20% space occupied at birth (Takahara et al., 1986). This mesenchyme disappears by 1 year of age, although cavitation of the mastoid air spaces continues further for a few years. In contrast, children with congenital abnormalities are estimated to have more mesenchyme in the bulla at birth, with this tissue taking longer to disappear postnatally (Takahara and Sando, 1987). Retention of mesenchyme in the middle ear in patients has been linked to a high incidence of otitis media (Jaisinghani et al., 1999). The retained mesenchyme and links to otitis media shown here also mirror the defects observed in the FBXO11J/+ mutant mice, where the mesenchyme fails to retract and leads to fibrous adhesions and dystrophic mineralization (Del-Pozo et al., 2019). Such mesenchymal defects, however, are not a characteristic of all mouse models of OM, such as the *Mecom, Junbo,* and *Tbx1* het mice, where cavitation precedes normally but defects are associated with changes to the innate immune system or eustachian tube function, respectively (Fuchs et al., 2015; Bhutta et al., 2017; Del-Pozo et al., 2019).

The angle of the eustachian tube was not significantly changed in the *Eya1* het mice. Similarly, ET defects have not been mentioned in BOR syndrome patients (Smith, 2018). However, other changes to the tube might be present. In *Tbx1* mice, the ET is morphologically normal but the muscles that drive its action are reduced in size, leading to defects in clearance (Fuchs et al., 2015). The *Eya1* and *Tbx1* null mutants share similar defects in the cardiac and craniofacial regions (Guo et al., 2011), with *Eya1* located upstream of *Tbx1* in the inner ear (Friedman et al., 2005). Together, these similarities highlight that it is possible that the ET muscles are also compromised in the het mice, which is a potential area to explore in the future. The expression of *Eya1* and *Eya4* shows some overlap in the eustachian tube epithelium, suggesting that some compensation may occur in single mutants. In keeping with this, *Eya1* and *Eya4* share a sequence homology of 71%, compared to 67% for *Eya2* and 48% for *Eya3.* Mild hearing defects have also been observed in *Eya2* mutant mice (Zhang et al., 2021). *Eya2* expression has only been studied prenatally but has very specific expression in cranial ganglia and the inner ear (Zhang et al., 2021). *Eya2* and *Eya1* have redundant functions in hypaxial myogenesis, highlighting the potential for compensation in other tissues (Grifone et al., 2007). *Eya3* has recently been linked to oculo-auriculo-vertebral spectrum, with patients displaying external ear defects and having both conductive and sensorineural hearing loss (Tingaud-Sequeira et al., 2021). The *Eyas* as a group, therefore, appear to have both distinct and overlapping functions in the ear.

The phenotype was often unilateral, suggesting some stochastic changes or environmental impact. Treatment with enrofloxacin has previously been shown to alleviate otitis externa in mice (Del-Pozo et al., 2022), but had no effect on the incidence of OM in the *Eya1* heterozygous mice. Antibiotics delivered to mice in drinking water have been shown to have poor efficacy against bacterial pathogens (Marx et al., 2014). It is, therefore, questionable whether this is a viable method to alleviate the signs of OM in mice. As an alternative, the mice were rederived and housed in a clean facility. At early postnatal stages, the mice showed a similar defect in cavitation, with retained mesenchyme in the attic and along the promontory, but this was resolved at later stages, and no OM was observed for 6 weeks. This is an important result as it shows that delayed mesenchymal retraction in mice with small auditory bullas can be rescued in the absence of OM. The presence of OM, therefore, appears to halt cavitation, causing permanent retention of mesenchyme in the ear. Retained mesenchyme thus sensitizes the ear to OM, while OM prevents any further regression of the mesenchyme from occurring. The presence of OM in the conventionally housed colony suggests that exposure to high levels of microbes may promote OM in sensitized ears. A comparison of the microbe burden on the two sites would therefore be an interesting addition to this study.

*Eya1* was shown to be expressed in postnatal tissues, but the changes observed in the epithelium were linked to the consequence of otitis media rather than a direct effect of a reduction in *Eya1* expression. As observed in other models, enhanced proliferation of the epithelium was observed with defects in the secretory cells and cilia. Moreover, defects in the localization of K5 cells were noted, with these cells losing their basal restriction in the polyps on the promontory. This is in accordance with the idea that it is the basal cells that undergo hyperplasia (Tos, 1980). K5 cells have previously been shown to increase in number but retained their basal location in *Tbx1* het mice with otitis media (Tucker et al., 2018). In contrast to the *Eya1* mice, the *Tbx1* mice do not have cavitation defects, suggesting that the K5 positive polyps in the *Eya1* mice might be associated with the retained mesenchyme. Alternatively, the stage of the disease may determine the extent to which the basal cells lose their normally restricted localization.

A novel phenotype change was noted in the expression of p63 in ears with early signs of OM. p63 was found to be at low levels in the middle ear of mouse on P21 but was significantly upregulated in the ears with signs of OM. In skin, *p63* controls the balance between proliferation and differentiation, with knockout mice having failed stratification (Koster et al., 2004). Importantly, ectopic expression of p63 alone is sufficient to drive stratification in simple epithelium, with p63 sitting upstream of other basal markers such as K5 (Sethi et al., 2014). In normal middle ear postnatal development, p63, therefore, turns off to maintain the simple epithelial structure observed in the ear, but in cases of OM, p63 is turned on/or maintained, causing the characteristic hyperplasia of OM. Interestingly, p63 has previously been shown to be expressed in a middle ear culture model (Mather et al., 2021). It is therefore possible that in reconstituted epithelium, the basal cells might revert back to a more primitive state and turn on p63. Given the potential role of p63 in driving the epithelial response to OM, it might be a good target for reducing the impact of OM on the ear.

The *Eya1* het mutants had significantly smaller auditory bulla compared to littermate controls, in accordance with other models of OM where the size of the bulla is dysmorphic or reduced in size (Richter et al., 2010; Kiyama et al., 2018). This is presumable owing to defects during embryonic development. For example, the small bullas in a mouse model of Treacher Collins syndrome were shown as a result of early neural crest defects associated with reduced postnatal proliferation (Richter et al., 2010). *Eya1* has been shown to be expressed in a wide variety of tissues (Xu et al., 1997; Kalatzis et al., 1998; Amin and Tucker, 2006), and how earlier expression of *Eya1* might drive changes in bulla size is an interesting area for future exploration.

Our study highlights the importance of auditory bulla size and shape in driving the susceptibility to OM, fitting with the described bulla defects observed in BOR patients (Smith, 2018). Early scans of syndromic patients would, therefore, be able to potentially identify vulnerable patients, particularly those with high levels of retained mesenchyme. If otitis media can be prevented at the key stages when the mesenchyme is still retracting, it might be possible to avoid permanent retention of mesenchymal tissue in the ear.

## Data Availability

The original microCT datasets are publicly available at Face Base (https://www.facebase.org/), record ID 1Q-X80R.

## Supplementary material

SUPPLEMENTARY FIGURE S1 Extent of retained mesenchyme during cavitation varies between mutants. *Eya1* heterozygous mice exhibit broad variation in the level of cavitation at P11 compared to wild-type littermates **(A)**. In some animals, cavitation appeared to progress normally **(B)**, while some exhibit retained mesenchyme on the promontory **(C,D)**, and others had mesenchyme retained in large swaths in the hypotympanum **(E,F)**. All scale bars equal 200 µm.

SUPPLEMENTARY FIGURE S2 No clear sex differences in cavitation or ET angle. Comparison of bulla volume (**(A)**, left hand ears), bulla bubble size (**(B)**, all ears) and ET angle (**(C)**, all ears) in female and male mice from the same litter.

SUPPLEMENTARY FIGURE S3
*Eya1* is expressed in the inner ear at P11. RNAscope multiplex fluorescence assay for *Ecad* in red (‘) and *Eya1* (‘’) in green. Inner ear. **(B,C)** higher magnification of boxes in **(A)**. *Eya1* was expressed in the hair cells in the organ of corti. N =2, Scale Bars: **(A)** = 200 µm; **(B,C)** = 50 µm.

SUPPLEMENTARY FIGURE S4 Increased proliferation of the middle ear epithelium during otitis media. Immunofluorescence for PCNA (red) at p21 in WT and *Eya1* heterozygous **(D–I)** middle ears with **(D–F)** and without **(G–I)** Otitis media (OM). **(A–C)** Control littermate with no proliferation evident in the epithelium of the middle ear. **(D–F)** The epithelium is proliferative (red) (arrows note proliferating epithelial cells) in cases of OM all across the middle ear, including the hypotympanum **(D)**, Promontory **(E)** and Attic **(F)**. **(G–I)** The contralateral ear of this mouse showed no evidence of OM, and very limited proliferation in comparison to the ear with otitis media (OM). Dotted line underlines the epithelium. N=2 Eya+/+; N=3 Eya1+/−. Scale Bars=50 µm.

SUPPLEMENTARY FIGURE S5 Rescue of OM after rederivation to a clean environment. When transferred to a clean colony, *Eya1* heterozygotes are able to complete cavitation of the middle ear so that they are indistinguishable from wild-type littermates **(A)**. Arrows indicate areas of the attic that are completely free from mesenchyme. No mesenchyme in *N* = 21/22 Eya1 Het ears. All scale bars set to 200 µm.
