# Supplementary figures and images for: The interconnected relationships between middle ear bulla size, cavitation defects, and chronic otitis media revealed in a syndromic mouse model
Source: Front Genet. 2022 Oct 10;13:933416. doi: 10.3389/fgene.2022.933416 (PMC9590451; doi:10.3389/fgene.2022.933416)

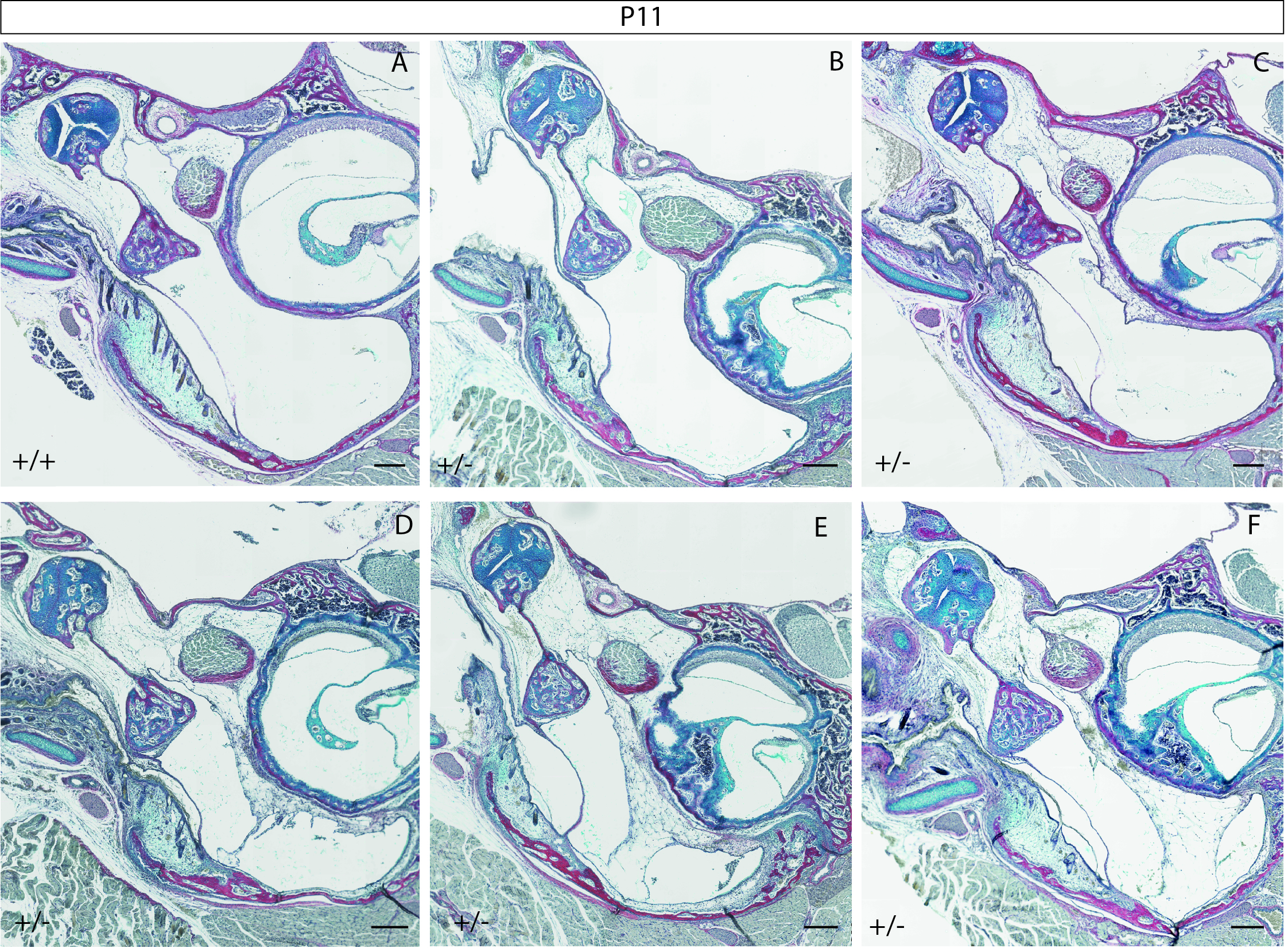

Supplement: Supplementary file 1 [file DataSheet1.zip › Supplementary files Fons 2022/Supplementary Fig 1.jpg]

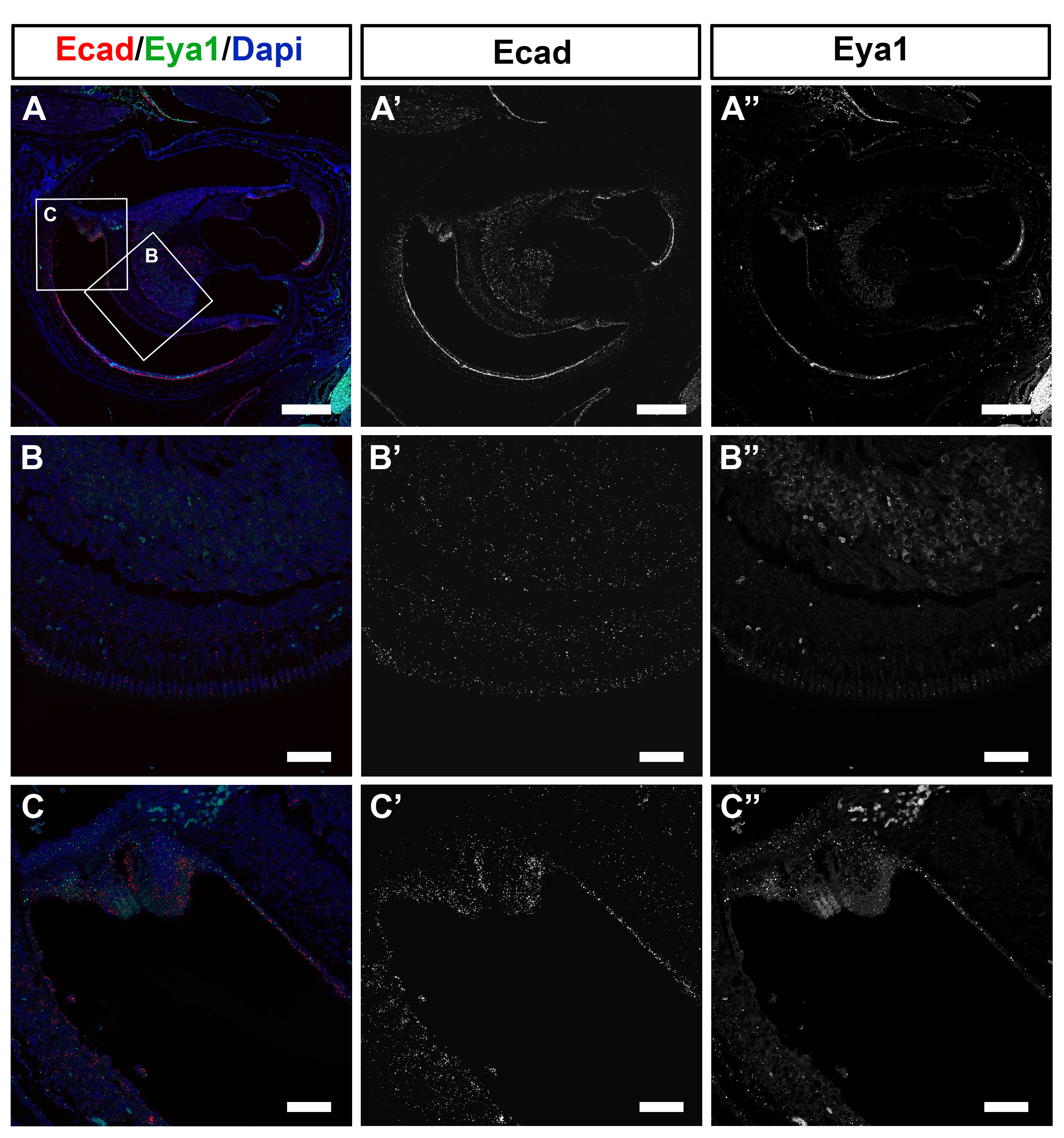

Supplement: Supplementary file 1 [file DataSheet1.zip › Supplementary files Fons 2022/Supplementary Fig 3.jpg]

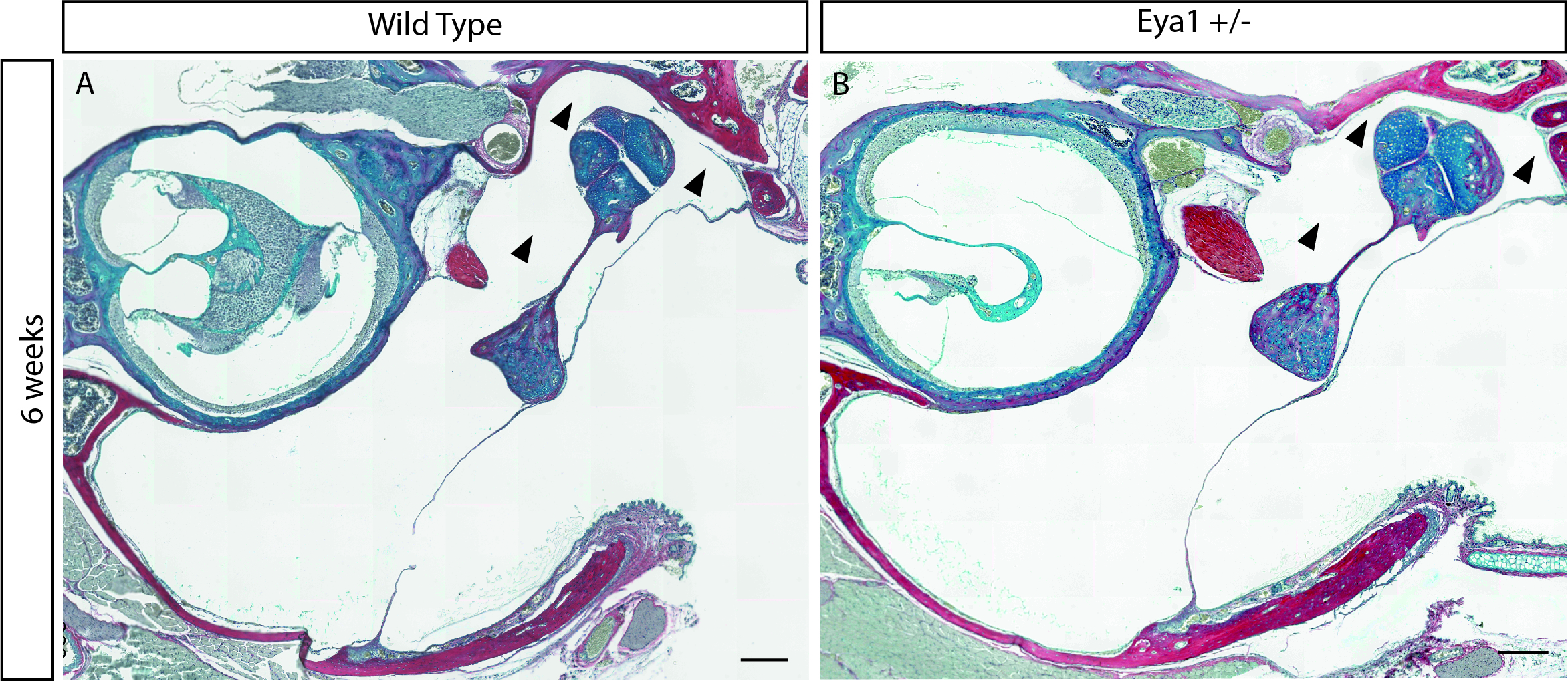

Supplement: Supplementary file 1 [file DataSheet1.zip › Supplementary files Fons 2022/Supplementary Fig 5.jpg]

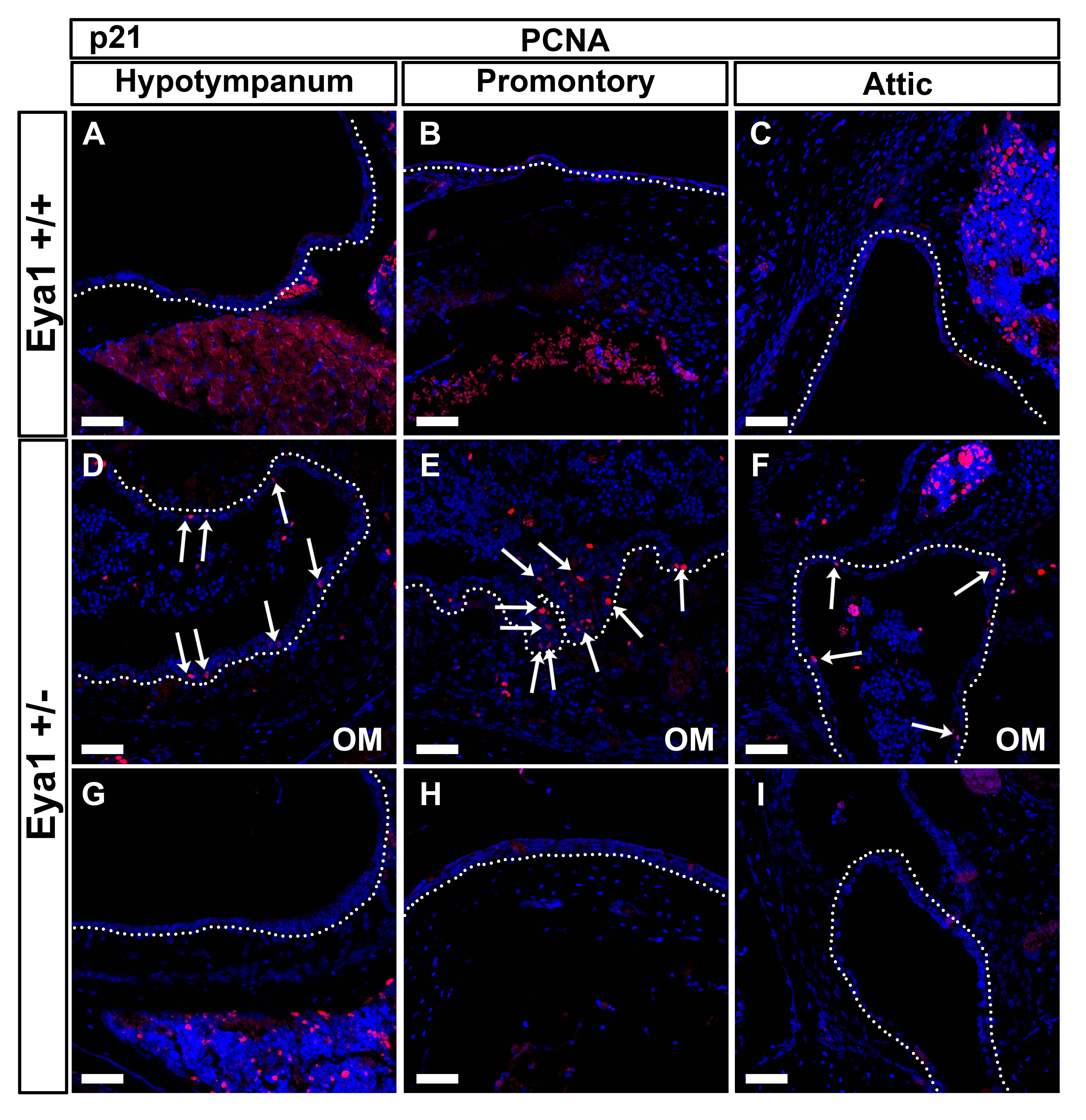

Supplement: Supplementary file 1 [file DataSheet1.zip › Supplementary files Fons 2022/Supplementary Fig 4.jpg]

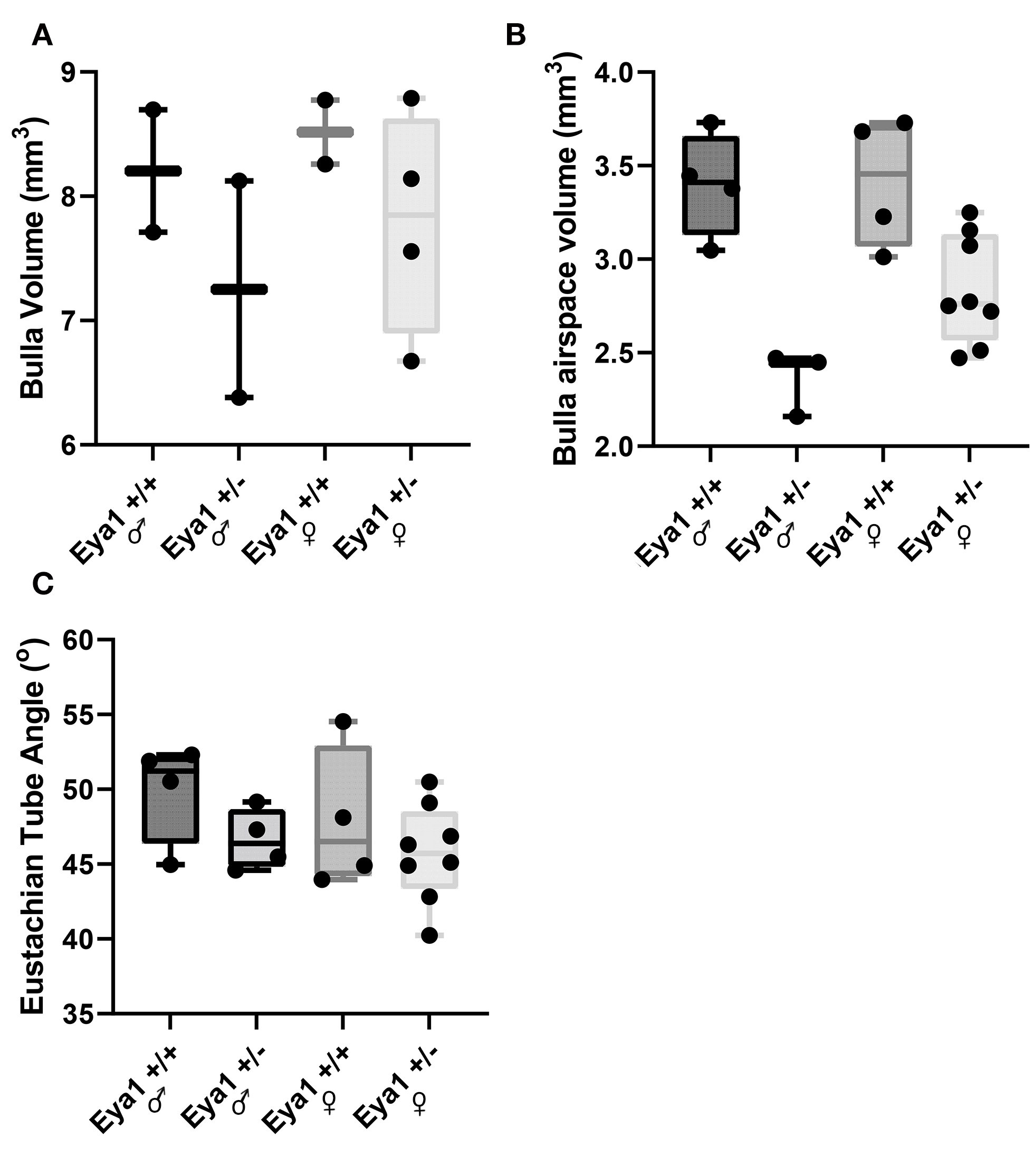

Supplement: Supplementary file 1 [file DataSheet1.zip › Supplementary files Fons 2022/Supplementary Fig 2.tif]
